# Paroxysmal Supraventricular Tachycardia Treatment: Evaluating the Evidence for Established and Newly Approved Options

**DOI:** 10.1016/j.jacadv.2026.103016

**Published:** 2026-07-22

**Authors:** Todd C. Villines, Narendra Singh, Anne-Sophie Lacharite-Roberge, Jonathan C. Hsu, Farhad Rafii, A.J. Blood

**Affiliations:** a University of Virginia; Division of Cardiovascular Medicine, Charlottesville, Virginia, USA; b NSC Cardiology; Division of Cardiology, Medical College of Georgia, Augusta University, Atlanta, Georgia, USA; c University of California at San Diego; Division of Cardiology, San Diego, California, USA; d Interventional Cardiology Medical Group, West Hills, California, USA; e Brigham and Women’s Hospital, Boston, Massachusetts, USA

**Keywords:** catheter ablation, etripamil, paroxysmal supraventricular tachycardia, pill-in-the-pocket, PSVT, vagal maneuvers

## Abstract

Paroxysmal supraventricular tachycardia (PSVT) imposes a substantial burden on patients and health care systems. Despite available treatments, a notable gap exists between evidence-based medicine and real-world practice. Acute treatments such as intravenous adenosine require administration in clinical settings. At-home vagal maneuvers are simple and cost-effective yet are often less effective than those administered by health care providers. The pill-in-the-pocket strategy, defined as patient-administered beta blocker or calcium-channel blocker therapy, is limited by delayed onset of action and risk of side-effects, and is not supported by robust clinical evidence or guidelines. Approved in late 2025, etripamil nasal spray represents a promising advancement that may provide a safe, effective, self-administered therapy used outside of health care setting. In this review, we examine the existing evidence and practical limitations of current PSVT treatments and discuss the potential clinical implications of etripamil as a novel, rapidly acting, self-administered therapy for the termination of acute symptomatic PSVT.

## Highlights

• Current acute PSVT treatments remain clinical setting dependent and burdensome for patients and health care systems.
• Evidence supporting at-home “pill-in-the-pocket” PSVT therapy is limited, outdated, and poorly generalizable.
• Emerging PSVT therapies may reduce emergency care use while improving outpatient management and quality of life.

## Central Illustration

**Figure:**
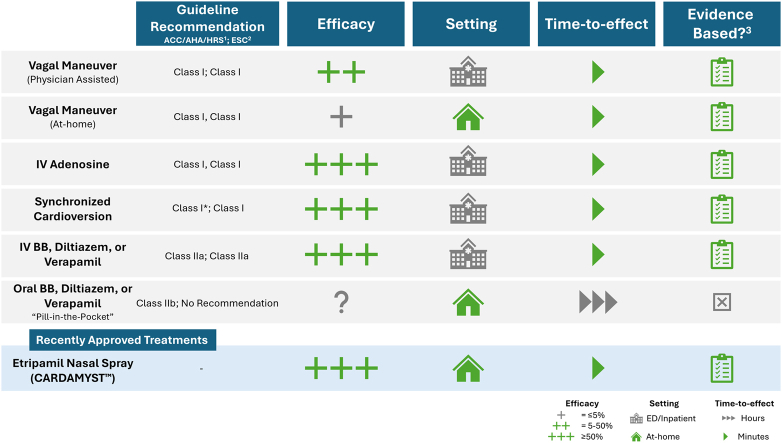

Paroxysmal supraventricular tachycardia (PSVT) is an arrhythmia characterized by regular and rapid tachycardia typically >100 to 250 bpm, with abrupt episodic onset and termination.^1^, ^2^, ^3^ PSVT affects ∼1 in 300 people in the United States, contributing to ∼250,000 emergency department (ED) and hospital visits annually.^4^^,^^5^ PSVT management often requires urgent health care visits resulting in increased costs and patient inconvenience.^1^^,^^2^ PSVT treatments include in- and out-patient and at-home physiological and pharmacological treatments, as well as procedural approaches (ie, ablation).^1^^,^^2^

PSVT encompasses atrioventricular (AV) nodal dependent arrhythmias such as AV nodal reentrant tachycardia (AVNRT) and AV reentrant tachycardia (AVRT), which comprise ∼90% of PSVTs.^1^, ^2^, ^3^ Patients commonly experience palpitations, shortness of breath, lightheadedness, fatigue, chest pressure/pain, and presyncope/syncope.^1^^,^^2^^,^^6^ Symptoms impair quality of life (QoL), particularly when episodes are frequent, prolonged, or disruptive to daily activities such as work, exercise, or travel.^1^^,^^7^ Diagnosis is delayed, often by years, due to PSVT’s episodic nature and limitations in capturing events required for definitive diagnosis with standard electrocardiography (ECG).^1^^,^^7^ Up to 50% of patients are initially misdiagnosed, for example, with anxiety or panic disorders.^8^ Projections from 2018 census data estimate the prevalence and incidence of PSVT in the United States to be 2.06 million and 306,000 cases, respectively.^4^ PSVT is associated with significantly higher health care resource utilization including ED and physician office visits, hospitalizations, and diagnostic testing.^9^, ^10^, ^11^, ^12^

This review evaluates the data for established and newly approved PSVT treatment options, and highlights the need for effective, evidence-based outpatient approaches to empower patients to treat acute episodes of PSVT (Central Illustration).

**Central Illustration:**
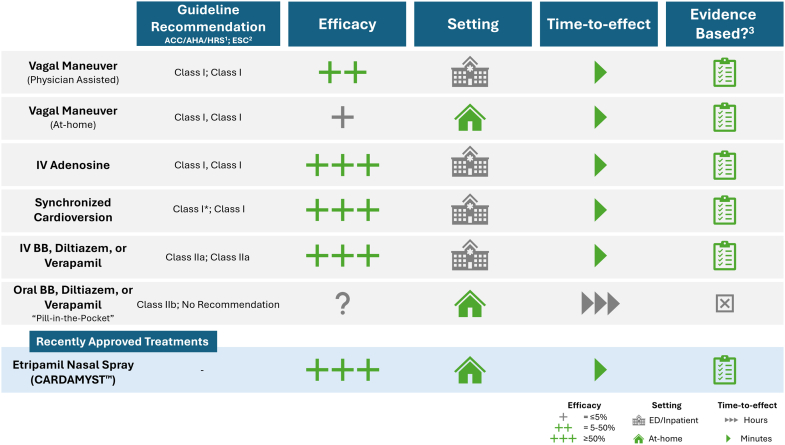
Overview of Acute Treatments for Paroxysmal Supraventricular Tachycardia Comparing PSVT treatment options by guideline recommendations, efficacy, treatment setting, time-to-effect, and evidence base. ^1^ACC/AHA/HRS guidelines, 2015. ^2^ESC Guidelines, ^3^Availability of applicable supporting clinical research for indicated use. ∗Class I when vagal maneuvers or adenosine are ineffective or not feasible. ACC/AHA/HRS = American College of Cardiology/American Heart Association/Heart Rhythm Society; BB = beta blocker; ESC = European Society of Cardiology; IV = intravenous.

## Global management guidelines for PSVT

PSVT management is primarily informed by the 2015 American College of Cardiology/American Heart Association/Heart Rhythm Society (ACC/AHA/HRS)^1^ guideline and the 2019 European Society of Cardiology (ESC) guideline.^13^ Both guidelines are broadly aligned in their recommendations for the acute treatment of PSVT; however, several notable differences exist. In hemodynamically stable patients, both guidelines recommend vagal maneuvers (VMs) as initial therapy, followed by intravenous (IV) adenosine when VMs are ineffective. IV calcium channel blockers (CCBs) or beta blockers (BBs) may also be used in selected patients when adenosine is ineffective or contraindicated, whereas synchronized cardioversion is reserved for hemodynamically unstable patients.

Despite this broad consistency, current guideline-directed acute therapy is largely centered on interventions administered in monitored health care settings, particularly IV adenosine, IV AV nodal blocking agents, or electrical cardioversion. The ACC/AHA/HRS guideline includes discussion of a “pill-in-the-pocket” approach using self-administered oral pharmacologic therapy in carefully selected patients with infrequent, well-tolerated PSVT episodes; however, this approach is limited by delayed onset of action and is not included as a formal recommendation in the ESC guideline. Neither guideline provides a widely adopted, rapid-acting, patient-administered acute therapy specifically designed to terminate AV nodal–dependent PSVT outside the health care setting.

Compared with ACC/AHA/HRS, the ESC guideline reflects a more restrictive approach to pharmacologic therapies and adopts a more proactive approach toward electrophysiologic risk stratification and prophylactic ablation. In contrast, the ACC/AHA/HRS guideline places comparatively greater emphasis on individualized decision-making and shared discussion regarding invasive intervention. The ESC guideline also places greater emphasis on minimizing antiarrhythmic drug exposure during pregnancy, particularly during the first trimester.

In addition to the ACC/AHA/HRS and ESC guidelines, several international and regional societies have published consensus documents or guidelines that inform the management of PSVT. Across regions, there is consensus supporting VMs and adenosine as first-line therapies for the acute treatment of PSVT, with synchronized cardioversion reserved for hemodynamically unstable patients. However, these approaches require either successful patient-performed VMs or treatment in a health care setting. A gap remains for a rapid, patient administered therapy that can terminate AV nodal-dependent PSVT outside of the health care setting. Regional differences generally relate to the emphasis placed on catheter ablation, pharmacologic therapy, and electrophysiologic risk stratification. Japanese and Asia-Pacific guidance documents reflect contemporary electrophysiology (EP)-focused management strategies, and Canadian and many Latin American practices commonly incorporate North American treatment paradigms derived from the ACC/AHA/HRS guideline, including broader discussion of pharmacologic management. Despite regional variation in implementation and health care infrastructure, international PSVT management guidance documents are generally consistent.

## Treatments for acute PSVT

### Vagal maneuvers

VMs have a Class I (strong) recommendation in the 2015 ACC/AHA/HRS guidelines.^1^ VMs increase vagal parasympathetic tone to slow conduction through and block the AV node and include carotid sinus massage (CSM), standard Valsalva maneuver (SVM), modified Valsalva maneuver (MVM), and diving reflex.^1^^,^^14^, ^15^, ^16^ CSM requires application of firm pressure to the carotid sinus to trigger vagal stimulation but carries stroke risk in older adults.^14^ SVM and MVM use forced exhalation achieved by blowing into a syringe, although the modified version includes leg elevation, making the maneuver difficult for some patients.^14^ The diving reflex involves breath-holding and cold exposure to slow AV nodal conduction.^14^ VM termination rates vary from 4% to >50%, and effectiveness remains unknown as most studies are health care provider-assisted with limited patient-performed maneuver data^14^^,^^17^, ^18^, ^19^, ^20^, ^21^ (Table 1). MVM efficacy, ∼40%-50%, is superior to SVM and CSM in PSVT termination, although all studies were ED conducted, which may not reflect the at-home success rate.^14^^,^^17^, ^18^, ^19^ The NODE-301 and RAPID trials observed 4% to 6% at-home VM conversion rates despite participants having been pretrained in the maneuvers^20^^,^^21^ (Table 1).

**Table 1:** Options for Acute Management of AV-Nodal Dependent PSVT

Treatment	Efficacy	Setting	Time to Effect	Side Effects	Guideline Recommendation^1^
Vagal maneuvers (eg, Valsalva, carotid sinus massage)	4%->50%^14^^,^^17^, ^18^, ^19^^,^^21^	Outpatient (self-directed)/Inpatient (physician directed)	Minutes	Minimal	Class I (strong), LOE B-R, ACC/AHA/HRS, 2015; Class I, LOE B, ESC 2019
Intravenous (IV) adenosine	85%-96%^22^, ^23^, ^24^, ^25^, ^26^	Acute health care setting	Seconds	Impending doom, bradycardia, dyspnea	Class I (strong) LOE B-R, ACC/AHA/HRS, 2015); Class I, LOE B, ESC, 2019
IV non-dihydropyridine calcium channel blockers (CCBs)	IV diltiazem: 82%-100%^27^ IV verapamil: 70%-80%^28^	Acute health care setting	Minutes	Hypotension, bradycardia, fatigue	Class IIa (moderate), LOE B-R, ACC/AHA/HRS, 2015; Class IIa, LOE B, ESC, 2019
IV beta blockers (BB)	IV esmolol: 84%^29^	Acute health care setting	Minutes	Hypotension, bradycardia, fatigue	Class IIa (moderate), LOE B-R, ACC/AHA/HRS, 2015; Class IIa, LOE C, ESC, 2019
Oral pill-in-the-pocket (PiP) monotherapy^a^	N/A	Outpatient (tablet)	Oral CCB and BB: ∼1-2 h onset	Hypotension, bradycardia, fatigue	Class IIb (weak), LOE C-LD, ACC/AHA/HRS, 2015; omitted, ESC, 2019
Crushed oral PiP combination therapy^a^	Oral diltiazem with oral Propranolol: 71% within 30 min^30^, 94% within 2 h^31^ Oral flecainide: 61% within 2 h^31^	Outpatient (patient self-crushed tablets)	∼27-32 min	Hypotension, bradycardia, fatigue	There is currently no guideline recommendation supporting this approach
Intranasal CCB (etripamil)	64% conversion to SR within 30 min^21^	Outpatient (nasal spray)	∼7 min (Tmax)	Nasal discomfort, congestion, rhinorrhea, throat irritation, epistaxis	N/A, newly available after recent US FDA approval (2025)^32^

ACC/AHA/HRS = American College of Cardiology/American Heart Association/Heart Rhythm Society; B-R = level B, randomized; C-LD = level C, limited data; ESC = European Society of Cardiology; FDA = Food and Drug Administration; LOE = level of evidence; N/A = not applicable; PSVT = paroxysmal supraventricular tachycardia; SR = sinus rhythm; Tmax = time to maximum concentration; US = United States.

a There are no data specifically studying the effect of oral beta blocker monotherapy for the acute termination of AVNRT. However, 2 studies have demonstrated success with the combination of oral diltiazem and propranolol to terminate AVNRT or AVRT.

### IV Adenosine, IV calcium channel blockers, and IV beta blockers

IV adenosine holds a Class I (strong) recommendation in the 2015 ACC/AHA/HRS guidelines.^1^ Adenosine, a short-acting antiarrhythmic with vasodilatory properties, exerts its therapeutic effect by activating adenosine A1 receptors in the heart, inducing a transient AV nodal block.^22^^,^^23^ This interrupts reentrant circuits underlying most cases of PSVT, restoring sinus rhythm (SR) with reported success rates of 85% to 96%^1^^,^^24^, ^25^, ^33^, ^34^ (Table 1). Its short half-life (seconds) necessitates rapid IV push administration followed by a saline flush to optimize delivery. Common adverse events (AEs) include transient AV block and asystole, flushing, chest pain, hypotension, dyspnea, atrial fibrillation (AF), bronchospasm, or coronary steal, with typically transient effects due to adenosine’s short half-life.^1^^,^^35^ Adenosine acts on receptors in vascular and neural tissue, causing vasodilation, bronchoconstriction, and stimulation of sensory nerve endings, which can trigger a feeling of panic or unease. Patients commonly describe a profound sense of “impending doom” immediately postinjection of adenosine.^35^ These reactions, although brief, are distressing and limit patient confidence for future treatment.

For hemodynamically stable patients in whom adenosine fails, IV CCBs diltiazem and IV verapamil carry a Class IIa (moderate) recommendation in the 2015 ACC/AHA/HRS guidelines.^1^ Both IV diltiazem and IV verapamil slow AVN conduction, prolong AVN refractoriness, and have shown superiority over placebo in conversion of PSVT to SR;^27^^,^^28^ and have been shown to terminate SVT in 64% to 98% of patients.^1^^,^^33^ Hypotension, worsening heart failure in patients with pre-existing ventricular dysfunction, and bradycardia are frequently documented potential AEs of IV diltiazem and IV verapamil^1^ (Table 1).

Like IV CCBs, IV BBs have a Class IIa (moderate) recommendation for the acute treatment of SVT in the 2015 ACC/AHA/HRS Guidelines postadenosine failure.^1^ Supporting data for their efficacy in terminating PSVT are limited.^1^ Hypotension, worsening heart failure, bronchospasm, and bradycardia are most common potential AEs^1^ (Table 1).

### Catheter ablation

Catheter ablation is a Class I (strong) recommendation for patients with symptomatic and recurrent SVT per the 2015 ACC/AHA/HRS guidelines.^1^ Large registry studies report the success rates of catheter ablation to be >95% in the treatment of ongoing PSVT.^1^ Ablation is often the right choice for many patients as it is associated with positive long-term QoL outcomes.^36^ Despite its efficacy and low complication risk, only ∼15% of patients pursue an ablation due to the invasive nature of the procedure, wait times, reluctance, or regional procedural availability.^1^^,^^4^

## Pill-in-the-pocket for acute PSVT

### Overview

The customary pill-in-the-pocket (PiP) approach, defined as episodic, self-administered oral medications for acute PSVT termination, carries a Class IIb (weak) recommendation for the termination of acute episodes of AVNRT per the 2015 ACC/AHA/HRS guidelines^1^ (Table 1). Due to the lack of relevant PiP evidence, it is not possible to have uniform recommendations, highlighting a gap between evidence-based medicine and real-world practice. Self-administered oral non-dihydropyridine (DHP) CCBs (eg, diltiazem, verapamil), BBs (eg, metoprolol, propranolol), and class 1C antiarrhythmic drugs (eg, flecainide, propafenone) have been used off-label in patients with infrequent, well-tolerated episodes of PSVT;^1^ however, none of the common PiP agents are Food and Drug Administration–approved for as-needed acute PSVT termination.^1^ The specific guidelines’ descriptions of “infrequent” and “well-tolerated” PSVT episodes imply limited applicability for those with modestly frequent or severe recurrences. Utility is also limited simply because of delays in absorption from oral administration and, relative to parenteral delivery, lower peak drug levels which may be needed for termination of PSVT.

The data informing PiP guideline recommendations for acute PSVT termination are both limited and outdated with small cohorts and methodologic shortcomings (Table 2). Reasonably, the 2015 ACC/AHA/HRS guidelines state that “oral BBs, diltiazem, or verapamil may be reasonable for acute treatment in hemodynamically stable patients with AVNRT” and “overall, there are no data specifically studying the effect of oral BB monotherapy for the acute termination of AVNRT.” However, 2 studies (Yeh et al; Alboni et al) have demonstrated success with the combination of oral diltiazem and propranolol to terminate AVNRT or AVRT. Oral BBs have an excellent safety profile, and administration (particularly in patients without IV access) can be performed in conjunction with VMs”.^1^ Metoprolol, flecainide, and propafenone receive no guidelines mention for acute SVT management; for acute management of atrial flutter or fibrillation, which SVT patients may also have, administration of type IC agents (flecainide and propafenone) should take into account guidance to use only after rate control has been achieved. The 2019 ESC SVT guidelines have omitted the use of PiP.^13^

**Table 2:** Literature Summary of Pill-in-the-Pocket for Acute Treatment of PSVT

First Author, Year	Trial Design and Population	Drugs and Dosing	Efficacy	Setting and Follow-Up	Adverse Events^a^	Limitations
Yeh et al, 1985^30^	Randomized, controlled (n = 15), patients with induced PSVT	Crushed tablets, combination of oral diltiazem 120 mg + propranolol 160 mg	Conversion within 4 h: 93% vs 27% (*P* < 0.001); mean time to conversion 27 ± 15 min (active) vs 164 ± 89 min (placebo); 71% converted <30 min	EP lab (controlled environment); follow-up 5.6 months with at-home single-dose use	Dizziness, nausea, diaphoresis (n = 7); hypotension^4^; transient AV block^1^; bradycardia^1^	Small sample, induced (not spontaneous) PSVT; crushed tablets; combination therapy
Rose et al, 1986^37^	Randomized, controlled (n = 12), patients with induced PSVT	Crushed tablets, combination of oral pindolol 20 mg + verapamil 120 mg	Conversion within 240 min: 75% vs 0%; mean time to termination 28 ± 8 min vs 168 ± 20 min (*P* < 0.001)	EP lab (controlled environment); no outpatient phase	Polyuria (n = 6); severe palpitations^3^; dizziness^2^; transient AV block^2^; lightheadedness^1^	Small sample, induced (not spontaneous) PSVT; crushed tablets; combination therapy
Alboni et al, 2001^31^	Randomized, controlled (n = 37^b^), patients with induced PSVT	Crushed tablets, combination of oral flecainide (∼3 mg/kg), or diltiazem 120 mg + propranolol 80 mg	Conversion within 2 h: 94% D/P, 61% F, 52% P; mean time 32 ± 22 min (D/P) vs 74 ± 37 min (F); during 17 ± 12 mo follow-up, 81% D/P, 80% F converted within 2 h	EP lab (controlled environment); followed by home self-administration (long-term follow-up)	Mild nausea/headache/sweating (n = 8; 3 D/P, 5F); bradycardia (n = 4; 3 D/P, 1 F); hypotension (n = 3; 1 D/P, 2F)	Small sample, induced (not spontaneous) PSVT; crushed tablets; combination therapy

AV = atrioventricular; D = diltiazem; EP = electrophysiology; F = flecainide; P = propranolol; other abbreviation as in Table 1.

a Among study drug treated patients (during the acute study phase if design included follow-up phase).

b 4 patients not included as SVT was noninducible or lasted <5 minutes, N = 33 were enrolled and separately underwent F, D/P, and placebo.

### Summary of PiP evidence

Yeh et al^30^ conducted a randomized, controlled, crossover trial in 15 patients with PSVT to evaluate the efficacy of a single crushed oral dose of diltiazem (120 mg) plus propranolol (160 mg) compared to placebo (Table 2). Sustained PSVT was induced in a cardiac electrophysiologic study on consecutive days, and study drug or placebo was administered 15 minutes after induction. Conversion to SR within 4 hours occurred in 93% (14/15) of patients after combined diltiazem/propranolol vs 27% (4/15) with placebo, with a mean time-to-conversion of 27 ± 15 minutes among converters. The combination significantly reduced ventricular rate at 30 minutes (179 ± 21 bpm vs 193 ± 21 bpm; *P* < 0.001) without significant hypotension. AEs included dizziness, nausea, diaphoresis, hypotension, transient AV block, and bradycardia. Despite high conversion efficacy, limitations include small sample size, high degree of medical monitoring in a cardiac catheterization lab setting, electronic induction (no study of spontaneous PSVT), use of crushed tablets, and combination therapy rather than monotherapy.

Alboni et al^31^ conducted a randomized, controlled trial in 37 patients with infrequent (≤5 per year), well-tolerated, ECG-documented PSVT requiring at least 1 annual ED visit and not receiving BBs, CCBs, or antiarrhythmics (Table 2). PSVT was induced on three consecutive days, and each patient (n = 33 included underwent each of the treatments) received placebo, crushed flecainide (∼3 mg/kg), or a combination of crushed diltiazem (120 mg) plus propranolol (80 mg). Conversion to SR within 2 hours occurred in 52% with placebo, 61% with flecainide, and 94% with diltiazem/propranolol (mean time-to-conversion: 77 ± 42, 74 ± 37, and 32 ± 22 minutes, respectively). AEs during EP testing included hypotension, bradycardia, mild nausea, headache, or sweating. Patients were later discharged on the most effective therapy and followed for ∼17 months. During follow-up, 81% of patients on diltiazem/propranolol and 80% on flecainide achieved conversion within 2 hours, with mean times of ∼40 minutes, and significantly reduced ED visits (9% vs 100% pre-enrollment; *P* < 0.0001). Most patients (73%) reported satisfaction and continued their assigned therapy. Treatment was discontinued during follow-up in 27% of patients; 1 syncope with trauma, 5 increased episode frequency; catheter ablation. The study concluded that a single, self-administered oral dose of diltiazem/propranolol or flecainide can be effective for managing infrequent, well-tolerated PSVT. Limitations include small sample size, reliance on induced arrhythmias under EP lab supervision, use of crushed tablets with combination therapy, and exclusion of patients on concurrent rate-control drugs.

Unlike Yeh et al and Alboni et al, Rose et al is not mentioned in the 2015 ACC/AHA guidelines; however, the study features notable similarities in design and methodology. The study by Rose et al^37^ was a randomized, controlled trial in 12 patients with recurrent, ECG-documented PSVT assessed the efficacy of a single oral dose of crushed pindolol (20 mg) plus verapamil (120 mg) vs placebo (Table 2). PSVT was induced on consecutive days and sustained for >30 minutes before administration of study drug or placebo, with patients observed for ≤240 minutes. In patients receiving pindolol/verapamil, ventricular rate significantly lowered (182 ± 5 bpm to 164 ± 7 bpm; *P* < 0.05) with no reported hypotension, and conversion to SR occurred in 75% (9/12), with a significantly shorter mean time-to-termination than placebo (28 ± 8 vs 168 ± 20 minutes; *P* < 0.001). AEs included polyuria, severe palpitations, dizziness, transient AV block, and lightheadedness. Plasma levels confirmed therapeutic absorption of both agents. The authors concluded that crushed combination pindolol/verapamil was effective for PSVT termination; however, the study’s limitations include small sample size, combination rather than monotherapy, use of crushed tablets, and lack of outpatient validation.

### Diminished PiP absorption during PSVT episodes

Acute oral therapy during PSVT is complicated by delays in gastric emptying due to sympathetic nervous system activation, which reduces drug absorption. For example, plasma levels after large oral doses of verapamil are significantly lower during PSVT compared with SR.^38^ Although this delay was demonstrated with verapamil, other oral agents are unlikely to meaningfully differ in gastric permeability. Studies of oral therapy absorption are often conducted in SR, when gastric motility is normal. The evidence supporting PiP use relies on crushed tablets to accelerate absorption.^30^, ^31^, ^37^ Furthermore, on PSVT episode termination, gastric motility can abruptly normalize and partially dissolved drug in the stomach may suddenly empty into the small intestine, leading to rapid absorption and potentially transient high plasma levels associated with hypotension or bradycardia.^38^

### PiP approach in modern practice

Contemporary PiP regimens vary widely across providers but commonly use oral BB monotherapy with uncrushed tablets (ie, metoprolol 25-50 mg), supplemented by oral antiarrhythmics (ie, flecainide 100-150 mg), as needed. Compared to fast acting IV treatments (minutes) which must be administered in a health care setting, oral BB or CCB treatment that can be taken outside of a health care setting has a relatively slow (∼1-2 hours) onset of effect (Table 1). Generic oral monotherapies (eg, metoprolol) can be relatively inexpensive, and despite the lack of evidence supporting their use, some patients report generally positive experiences with oral agents.

The presented PiP studies share common limitations that impact applicability to real-world cases, providing minimal evidence supporting practice. These include the study of induced, nonspontaneous PSVT that does not reflect real-world patient experiences, the use of crushed tablets and the combination therapy of multiple agents mixed and taken simultaneously (Table 2). Crushing or mixing oral tablets is cumbersome and impractical, particularly when a patient may be distressed during a PSVT episode, can lead to an increased risk of hypotension,^31^^,^^30^ and is not utilized in modern practice. The PiP regimen’s off-label status, limited, poorly applicable and outdated supporting evidence, and common side effects underscore the need for a reliable and portable option. The lack of self-administered treatments for acute PSVT termination has left patients reliant on the ED or ablation. To address these unmet needs, new evidence-based treatments given outside the health care setting for patients with PSVT must be considered.

## PSVT management in select populations

### PSVT presenting with wide-complex tachycardia

Wide-complex tachycardia (WCT) has notable diagnostic and management consideration in patients with suspected PSVT. WCT may reflect ventricular tachycardia, supraventricular tachycardia with aberrant conduction, or tachycardias involving accessory pathway conduction, including antidromic AVRT or AF with pre-excitation. In hemodynamically stable patients, diagnostic evaluation incorporates clinical history, 12-lead ECG characteristics, response to VMs, and when available, prior rhythm documentation. As diagnostic distinction can be challenging outside clinical settings, the guidelines recommend caution when treating undifferentiated WCT. AV nodal-blocking therapies, such as adenosine, non-DHP CCBs, and BBs, should not be used in patients with undifferentiated WCT or when pre-excited AF is suspected, as preferential conduction over an accessory pathway may result in hemodynamic instability or degeneration to ventricular fibrillation. Self-administered therapies intended for AV-nodal dependent PSVT are most appropriate for patients with an established diagnosis of regular, narrow-complex PSVT or who have received appropriate clinician instruction regarding treatment use. It is recommended that patients with a history of pre-excitation, prior episodes of unexplained WCT, or uncertainty regarding arrhythmia mechanism should be evaluated before considering self-administered AV nodal-blocking therapies.

### PSVT patients with congenital heart disease

In patients with congenital heart disease, SVT may arise from complex anatomy, prior surgical repairs, atrial scar-related re-entry, or accessory pathways, and management often requires individualized electrophysiologic evaluation. Catheter ablation can be more challenging in this population, and antiarrhythmic drug selection may be influenced by underlying structural abnormalities, ventricular function, or prior surgical interventions. Potential pharmacological intervention must be determined by the physician.

### Pregnant patients with PSVT

Pregnancy presents unique management considerations due to physiologic hemodynamic changes that can increase the frequency of arrhythmia, whereas fetal safety considerations influence decision-making. VMs and adenosine are generally considered first-line therapies for acute termination of hemodynamically stable PSVT during pregnancy, whereas use of CCBs, BBs, and invasive procedures typically requires individualized risk-benefit assessment, and management should involve shared decision-making with appropriate physician input.

## Recent advancement in acute PSVT treatment

### Etripamil nasal spray

In 2025, the Food and Drug Administration approved an intranasal CCB, etripamil (CARDAMYST; Milestone Pharmaceuticals USA Inc.), presenting a new option for self-administered termination of acute symptomatic PSVT episodes outside of the health care setting.^32^ Etripamil is a novel, non-DHP CCB that terminates PSVT through slowing AV nodal conduction and prolonging the AV nodal refractory period by blocking the slow inward calcium influx through L-type calcium channels^32^^,^^39^ (Table 1). Etripamil is formulated as a self-administered nasal spray with a rapid onset of action (T_max_ ≤7 minutes) and is inactivated by blood esterases with a terminal half-life of approximately 2.5 hours^39^ Its tolerability profile is favorable, with most AEs related to the route of administration (ie, nasal discomfort).^6^^,^^21^^,^^40^ As with other AV nodal-blocking therapies, etripamil is intended for AV nodal–dependent PSVT (eg, AVNRT or AVRT) and is not intended for undifferentiated WCT, ventricular tachycardia, or patients with pre-excitation syndromes (eg, Wolff-Parkinson-White with a manifest delta wave), where AV nodal blockade may be unsafe. Etripamil is supplied as two disposable nasal spray devices, each delivering 1 dose consisting of two sprays for a total of 70 mg, contained in a small plastic carrying case.^32^ Conveniently, etripamil can be stored at room temperature (68 °F-77 °F [20 °C-25 °C]), with excursions permitted from 59 °F to 86 °F [15 °C-30 °C]) and has a 36-month shelf life.

Etripamil has demonstrated consistent efficacy across trial phases and designs, significantly outperforming placebo and providing rapid PSVT symptom relief (median time-to-conversion: 18.5 minutes).^6^^,^^20^^,^^21^^,^^40^, ^41^, ^42^, ^43^ Across trials, the safety profile of etripamil is favorable and characterized by only predominantly mild, transient AEs localized to the nasal administration site, and low incidence of hypotension (0.4%) and syncope (0.2%).^6^^,^^20^^,^^21^^,^^40^, ^41^, ^42^^,^^44^ In the phase 2 NODE-1 trial conducted in an EP lab with induced PSVT (n = 104), a 70 mg dose achieved a conversion rate of 87% within 15 minutes compared to 35% for placebo (*P* < 0.001).^20^ Higher doses (ie, 140 mg) showed similar efficacy but increased AEs, establishing the 70 mg dose as optimal for phase 3 research.^20^ The phase 3 RAPID study achieved its primary efficacy endpoint of terminating PSVT with self-administered etripamil (70 mg, with optional symptom-based repeat 70 mg dose), HR 2.62, *P* < 0.001 (n = 184).^21^ Conversion of PSVT to SR occurred in 64.3% of patients at 30 minutes and 73.5% of patients at 60 minutes (placebo, 31.2% and 54.5%, respectively).^21^^,^^45^ The median time-to-conversion was 17.2 minutes with etripamil compared to 53.5 minutes with placebo.^21^ Although most patients achieved conversion with a single 70 mg dose, the RAPID study regimen incorporated an optional repeat dose if symptoms persisted for 10 minutes after the first dose. AEs occurring in ≥5% of patients were nasal discomfort (23%), nasal congestion (13%), and rhinorrhea (9%), and no events of syncope occurred. In the RAPID efficacy population, 64% of etripamil patients were using concomitant AV-nodal acting medications (β blockers or calcium-channel blockers). Among patients self-administering study drug, ∼9% had been enrolled after a failed ablation and ≥1 recurrence of PSVT. In a pooled secondary analysis of NODE-301 part 1 and RAPID, etripamil demonstrated a 39% relative risk reduction (relative risk: 0.61; 95% CI: 0.38-0.97; *P* = 0.04) in ED visits.^46^ NODE-302, an open label phase 3 extension, reported conversion within 30 minutes of etripamil to be 60.2% (median time-to-conversion, 15.5 minutes) among 188 PSVT episodes (n = 92).^41^ NODE-303, an open-label phase 3 study, evaluated self-administration in a real-world-like setting with single or repeat-dose etripamil for multiple PSVT episodes without a supervised test-dose.^40^ Conversion rates mirrored prior trials, with comparable safety, and recurrence rates postconversion were generally low in trials.^40^ Among 151 patients who self-treated at least 2 confirmed PSVT episodes, 71.5% converted in the first episode, and conversion was predictive of subsequent response, with 80.6% of those also converting in the second episode (chi-square = 9.67; *P* = 0.0019).^40^

The advantages of etripamil include on-demand treatment outside of a medical setting, rapid onset of action, short acting effects, and a favorable safety profile with AEs predominantly related to route of administration. Notably, a recent review article discussed how the combined advancement of wearable devices with the addition of etripamil signifies a paradigm shift in PSVT management where patients are now better equipped to self-treat their PSVT.^43^ Direct comparisons between the conversion rates of the EP-based PiP studies and the phase 3 etripamil studies are inappropriate due to fundamental differences in design (induced vs spontaneous PSVT), setting (supervised vs ambulatory), methodologies (crushed multiagent vs monotherapy spray), and cohorts (small, nonrepresentative vs diverse real-world). As clinicians may be inclined to draw comparisons, it is critical to emphasize that there have been no head-to-head studies of etripamil and oral CCBs or BBs.

### Vagal assist devices

Valsalva assist devices (VADs) are a new nonpharmacologic approach to potentially improve the efficacy of VMs for acute termination of PSVT. These handheld devices are designed to assist patients in achieving and maintaining a standardized target expiratory pressure (40 mmHg) which promotes an effective VM. These devices are aimed at addressing the limitations of standard syringe-based techniques, which have shown varied efficacy and may not obtain or hold an optimized expiratory pressure. Examples include VALSAVA-1 VAD (Shanghai TechBank Medical Technology Co, Ltd) and Valsa-Valve VAD (Meditech Systems Ltd), which is approved for clinical use in the UK and Europe as a CE-marked medical device.^1^, ^2^, ^3^ Advantages of this strategy include its relatively low cost, absence of risks related to systemic drug exposure, ease and safety of repeat use, and broad accessibility in healthcare and at-home settings.

A randomized clinical trial by Huang et al evaluated the VALSALVA-1 device in 212 patients with induced AVNRT or AVRT undergoing electrophysiologic study.^2^ Patients were randomized to standard VM or VALSALVA-1 device-assisted VM. After up to two maneuvers, conversion to SR within 1 minute occurred in 63.2% of patients in the device-assisted group compared with 29.2% in the standard VM group (OR 4.16; 95% CI, 2.36-7.47; *P* < 0.001). Following a single maneuver, cardioversion occurred in 45.3% vs 18.9%, respectively (OR 3.55; 95% CI, 1.94-6.72; *P* < 0.001). No serious AEs occurred, and minor adverse effects including lightheadedness, nausea, and shortness of breath were infrequent and similar between groups. These findings suggest that standardization of strain pressure may substantially improve VM efficacy under controlled conditions.

In contrast, the larger stepped-wedge cluster randomized trial by Appelboam et al, “EVADE-SVT (Evaluation of the prehospital use of a Valsalva Assist Device in the emergency treatment of SVT),” evaluated the use of the Valsa-Valve device in UK ambulance service.^47^ The study included 865 patients with suspected SVT treated in the out-of-hospital setting. The device and placebo were associated with similar overall cardioversion rates (38.8% and 37.5% respectively), but the device did not reduce hospital conveyance compared with standard care (89.3% vs 89.2%). There was a statistically significant reduction in ambulance “on-scene” time observed, as the device resulted in a mean reduction of 8.4 minutes (*P* = 0.04). Notably, an earlier randomized controlled feasibility study (EVADE) by Appleboam et al. found that VAD use was associated with cardioversion and nonconveyance in 4 (24%) and 2 (12%) participants respectively.^48^ Findings from both studies were generated in a real-world emergency medical ambulance services environment, yielding broadly appliable results; however, the authors concluded that they did not find sufficient evidence to support ongoing routine device availability in the ambulance service.

Together, these studies are inconclusive but suggest that VADs may find a role in support of acute PSVT management. Further research is needed to determine whether VADs enhance PSVT conversion rates compared with standard VMs, to more clearly identify the settings in which they provide the greatest value, and to assess whether they offer indirect benefits such as improved patient or provider convenience.

## Conclusions

PSVT imposes a substantial burden on patients and health care systems, with current acute management strategies largely confined to in-hospital or ED settings and characterized by variable efficacy. Catheter ablation remains a highly effective and durable preventive therapy; however, it is not pursued by many eligible patients and does not address the need for acute, episodic symptom control. A safe and effective self-administered therapy used outside the health care setting represents an important alternative for patients who are not ready to undergo ablation or who require interim symptom control while awaiting definitive therapy. Although historical PiP strategies may be appropriate for select individuals, their use is constrained by outdated and limited evidence, off-label administration, and clinically relevant adverse effects, including hypotension and AV block. In this context, intranasal etripamil represents a promising advance, offering the potential for rapid, self-directed termination of PSVT episodes. If integrated thoughtfully into clinical practice, this approach may improve QoL, reduce reliance on emergency care, and meaningfully expand patient-centered options for acute PSVT management.

## Funding support and author disclosures

This paper was funded by Milestone Pharmaceuticals. Dr Villines serves as a consultant (clinical trial advisor) for Abcentra, AngioInsight, Cleerly, HeartFlow, and Milestone; and reports research grants from the 10.13039/100000002National Institutes of Health. Dr Hsu has received fees/honoraria from 10.13039/501100005035Biotronik, 10.13039/100008897Janssen, 10.13039/100004374Medtronic, 10.13039/100002491Bristol-Myers Squibb, 10.13039/100001316Abbott Laboratories, Vektor Medical, 10.13039/100015345Zoll Medical, 10.13039/100004319Pfizer, 10.13039/100008497Boston Scientific, Biosense-Webster, i10.13039/100006983R10.13039/100006983hythm, Sanofi-Aventis, Altathera, Viz.Ai, Acutus, Milestone Pharmaceuticals; and grants from Biosense-Webster, and 10.13039/501100005035Biotronik. Dr Singh has received grants from Amgen, Astra Zeneca, Bayer, Boehringer Ingelheim, Cleveland Clinic- C5, Daiichi-Sankyo, Duke/DCRI, Eli Lilly, 10.13039/100008897Janssen, Johnson & Johnson, Kowa, Merck, Milestone Therapeutics, New Amsterdam, NHLBI, Novartis, Novo Nordisk, Oxford University, 10.13039/100004319Pfizer, Sanofi, Regeneron, and Timi Study Group; and has consulted for Amgen, AstraZeneca, Boehringer Ingelheim, CCRN, Esperion, 10.13039/100008897Janssen, Novo Nordisk, Sansar, and Sanofi. Dr Blood has received grants from Astra Zeneca, Boehringer Ingelheim, Novo Nordisk, Eli Lilly, General Electric Health, Merck, consulting fees from Astra Zeneca, Boehringer Ingelheim, Novo Nordisk, Corcept Therapeutics, Milestone Pharmaceuticals, NODE Health, Alnylam Pharma, Color Health, Medscape, Nference Inc., Walgreens Health, Withings, HelloHeart; holds equity in AIwithCare, Knownwell Health, Porter Health; and has a patent pending for Retrieval-augmented generation for medical data. All other authors have reported that they have no relationships relevant to the contents of this paper to disclose.
